# “Four-Step Procedure” of laparoscopic exploration for gastric cancer in West China Hospital: a retrospective observational analysis from a high-volume institution in China

**DOI:** 10.1007/s00464-018-6605-2

**Published:** 2018-11-26

**Authors:** Kai Liu, Xin-zu Chen, Wei-han Zhang, Dong-yang Zhang, Yi Luo, Yue Yu, Kun Yang, Shi-jie Yang, Xiao-long Chen, Li-fei Sun, Lin-yong Zhao, Zong-guang Zhou, Jian-kun Hu

**Affiliations:** 1 Department of Gastrointestinal Surgery and Laboratory of Gastric Cancer, State Key Laboratory of Biotherapy, West China Hospital, Collaborative Innovation Center for Biotherapy, Sichuan University, Chengdu, China; 2 West China School of Medicine, Sichuan University, Chengdu, China; 3 Department of Gastrointestinal Surgery and Laboratory of Digestive Surgery, State Key Laboratory of Biotherapy, West China Hospital, Collaborative Innovation Center for Biotherapy, Sichuan University, Chengdu, China

**Keywords:** Laparoscopic exploration, Four-Step, Staging, Gastric cancer

## Abstract

**Background:**

The preoperative work-up has limitations on finding peritoneal dissemination (PD) in gastric cancer patients. Laparoscopic exploration (LE) can discover radiographically occult PD, obtain accurate stage and avert futile laparotomy. The aim of our study was to introduce “Four-Step Procedure” LE in West China Hospital and further evaluate its safety and feasibility.

**Methods:**

We conducted a retrospective analysis on 165 patients from July 2016 to December 2017 who underwent “Four-Step Procedure” LE in gastrointestinal surgery department of West China Hospital. All the patients were diagnosed with gastric adenocarcinoma without explicit distant metastasis through Computed Tomography and/or Gastrointestinal Ultrasonography. Peritoneal lavage cytological examination (CY) was routinely performed during LE in our research. The “Four-Step” technical process of LE was introduced comprehensively. The clinicopathologic features and the presence of PD or CY at LE were analyzed, and the stratified analysis by cT and cN stages on the proportion of P1 and/or CY1 was also reported in this study.

**Results:**

Total of 165 patients accepted LE in our study, among these patients: 27 (16.4%) patients with P1 and/or CY1: 19 (11.5%) patients were found PD (P1), 17 (10.3%) patients with positive cytological examination (CY1) and 9 (3.6%) patients with P1Cy1. The stratified analysis by cT stage indicated that there was no P1 and/or Cy1 in cT1–cT2 stages, 1 (2.7%) patient with P1 and 1 (2.7%) with Cy1 in cT3 stage, 18 (20.0%) patients with P1 and 16 (17.8%) with Cy1 in cT4 stage. After LE, there were 74 (44.8%) patients underwent laparoscopic assistant gastrectomy, 25 (15.2%) patients with open gastrectomy, 50 (30.3%) patients with neoadjuvant chemotherapy and 16 (9.7%) patients with palliative chemotherapy and/or conversion therapy.

**Conclusion:**

“Four-Step Procedure” LE is reliable and feasible for gastric cancer. From our study, LE has unique superiority on ascertaining PD and cytological examination and LE should be recommended in cT4 stage gastric cancer before resection.

**Electronic supplementary material:**

The online version of this article (10.1007/s00464-018-6605-2) contains supplementary material, which is available to authorized users.

Gastric cancer is one of the most frequently diagnosed malignancies worldwide with an estimated 951,600 new cases and 723,100 deaths occurred every year [1]. More than 40% of gastric cancer cases occurring in China that resulted in a serious burden [2]. Gastric cancer with locoregionally advanced stage was overwhelming majority in China which causes a relative poor prognosis when compared with Japan and Korean [3–5]. The therapeutic model for advanced gastric cancer was also changing, and individualized and comprehensive therapy based on surgical resection is the mainstream for advanced gastric cancer [6].

The most important procedure for clinicians was to make and optimize treatment schemes for individuals to improve the prognosis of patients with gastric cancer. An exact preoperative staging was especially crucial for making therapeutic strategies and prognostic prediction. Despite the medical examinations for preoperative stage such as imaging, ultrasonic and endoscopic techniques had been significantly improved, and the detection of peritoneal metastases among gastric cancer was still with low sensitivity and specificity [6–8]. In addition, positive peritoneal lavage cytological findings had been verified to be an independent prognostic factor of gastric cancer that could only be executed during surgical procedures [9, 10].

Laparoscopic exploration (LE) was recommended for detecting occult peritoneal dissemination (PD), collecting lavage liquid for cytological examination with minimal invasion and avoiding redundant laparotomies by many guidelines [11–14]. LE was an effective way for detecting unsuspected PD and had superior over conventional imaging methods [15, 16]. A proportion of patients was found to have unsuspected, unresectable factors for tumor during LE. The cost-effective analysis also supported the validation of LE in gastric cancer patients [17]. However, there was still no consolidated inclusion criteria for LE in gastric cancer patients since the distribution of stage was very distinguished from each other [18, 19]. The criterion for the recruitment of patients with gastric cancer into LE was also lack of credible evidence in China.

Different centers might have different experience on the operational approach of LE in gastric cancer; in addition, there were very rare studies to introduce the manipulative flow or operative sequence of LE in gastric cancer briefly [20]. The purpose of this research was to introduce an experienced “Four-Step Procedure” flow of LE and further evaluated the safety and feasibility of LE in advanced gastric cancer patients from our institution.

## Materials and methods

### Ethical issues and patients

This study was based on the information gathered from the database of the Surgical Gastric Cancer Patient Registry of West China Hospital (WCH-SGCPR) under registration number WCH-SGCPR-2017-09. The establishment of this database was approved by the Research Ethics Committee of West China Hospital. Each patient in this study was adequately informed about their potentially therapeutic regimens by clinicians, and the final option was decided by patients; the informed consent was routinely endorsed before treatment.

Total of 343 patients with gastric cancer were admitted in a gastric cancer professional group of Gastrointestinal Surgery Department, West China Hospital from July 2016 to December 2017. There were 11 patients without surgical intervention since explicit distant metastasis detected in preoperative examination that left 165 cases in LE group and 167 patients in non-LE group. All the patients were assigned with individual treatment strategy in terms of their preoperative staging evaluated by high-quality CT scan and gastrointestinal ultrasonography (GUS).

### Preoperative diagnosis and definition of cancer staging

The preoperative work-up included gastroscope with biopsies, 256-detector row spiral computed tomography (256-row MDCT) of chest and abdomen, ultrasound of the gastrointestinal (GUS), barium radiography. Endoscopic ultrasound (EUS), magnetic resonance imaging (MRI) and positron emission tomography (PET) were not conventionally conducted in our institution. Tumor size was evaluated by endoscopy, GUS and MDCT. The assessment of tumor depth and the status of lymph were based on MDCT and GUS, and the maximum nodal diameter ≥ 1 cm was considered as suspicious nodal involvement.

All the results of preoperative work-up were inspected by gastric cancer specialists. The confirmation of clinical stage was consulted with imaging experts. The surgical approach was subsequently determined based on all above results. In our hospital, LE and laparoscopic gastrectomy were performed by experienced surgeons. The diagnostic and therapeutic regimens for gastric cancer in our study refer to NCCN guidelines and Japanese Gastric Cancer Treatment Guidelines 2014 [21, 22]. All the definitions including cancer staging, Borrmann types, clinicopathological features were chiefly in line accordance with 14th edition of the Japanese Classification of Gastric Carcinoma by JGCA [23].

### Definitions of peritoneal metastases and positive peritoneal cytology

Peritoneal metastases (P1) were defined as a condition in that patients had tumor deposits peritoneum, omentum or mesentery by pathological verification. The situation that free cancer cells were cytologically demonstrated in peritoneal lavage fluid was defined as CY1 [24]. The scope of PD was reclassified into four subtypes according to the latest Japanese Gastric Cancer Treatment Guidelines, the 5th edition: Level 1 (P1a), where PD detected in the peritoneum adjacent to tumor locally including lesser and greater omentum, anterior lobe of transverse mesocolon, capsula pancreatitis and spleen. Level 2 (P1b), where PD detected in the epigastric peritoneum (parietal peritoneum above umbilicus and visceral peritoneum above transverse colon). Level 3 (P1c), where PD detected in the hypogastric peritoneum. Level 4 (P1x), the distribution of PD was ambiguous, when the peritoneal metastases confirmed by preoperative imaging examination also belong to P1x [25].

### Inclusion and exclusion criteria for LE

The inclusion criteria included: (1) histologically confirm gastric adenocarcinoma; (2) patients with preoperative examination including high-quality enhanced CT scan and GUS; (3) patients with T3 or T4 gastric cancer with or without evidence of lymph node metastases on high-quality preoperative imaging; (4) patients with T1 or T2 gastric cancer on high-quality preoperative imaging that designed to underwent laparoscopic gastrectomy were also performed LE before routinely surgical resection.

The exclusion criteria of our study include: (1) other types of malignancies in stomach; (2) tumor with definite PD or other distant metastasis by preoperative inspections; (3) patients with gastric cancer underwent direct laparotomic gastrectomy or robotic assistant gastrectomy; (4) patients with history of abdominal surgery (except LC).

### Technique: the “Four-Step Procedure” LE of West China Hospital (supplementary material)

#### Step 1: anterior abdominal wall and surface of abdominal viscera

LE is performed under general anesthesia, patient is placed in the supine position with legs closed, and carbon dioxide (CO2) pneumoperitoneum is established with 12 mmHg. A 12-mm trocar was inserted through umbilical level was recommended. A 30° laparoscope was plugged into abdominal cavity to notarize whether the intestine was bruised below the puncture and check whether there was puncture injures.

1. The exploration of anterior abdominal wall was according to clockwise orientation and “O” route. The exploratory sequence was as follows: (1) Bilateral diaphragmatic dome, ligamentum teres hepatis and falciform ligament; (2) the left side of anterior abdominal wall; (3) hypogastric anterior abdominal wall; (4) the right side of anterior abdominal wall.
2. The exploration of surface of abdominal viscera was according to “S” route. The exploratory sequence was as follows: (1) the diaphragmatic surface of left liver lobe; (2) the diaphragmatic surface of right liver lobe; (3) the surface of transverse colon, the great omentum from left to right; (4) the left side of abdominal wall, the left paracolic sulcus and the surface of descending colon; (5) the inferior abdominal wall, the surface of small intestine; (6) the right side of abdominal wall, the right paracolic sulcus and the surface of ascending colon; (7) transfuse about 150 ml saline solution into hepatorenal recess and splenic recess, respectively.

#### Step 2: pelvic cavity and surface of abdominal viscera

Establish two operating poles on the right (or left) abdomen and plug into two 5-mm trocar. Then, alter the body posture to trendelenburg position (incline with 30° angle); (1) transfuse 200 ml saline solution into bilateral paracolic sulcus and pelvic floor. (2) Take the intestine out of the pelvic cavity by non-invasion grasping forcep and detect the bilateral fossa iliaca and bilateral accessories (for female); (3) suck and collect the peritoneal lavage for cytological examination. (4) Lift the fundus vesicae urinariae or fundus of uterus and pull the sigmoid to detect pelvic floor and peritoneal reflection; (5) if there were suspicious tumor deposit, protractor biopsy by laparoscopic scissors and hemostasis was performed by electrocautery. These specimens were sent to frozen biopsy immediately as well as permanent paraffin-embedded sections.

#### Step 3: the mesentery and the small intestine

Recover the posture to supine position or the dorsal elevated position (incline with 30° angle); (1) upturn the great omentum to supracolic region and erect the transverse colon by two nippers to detect the mesocolon transversum and the root of mesocolon transversum, the Treitz ligament and evaluate the penetrating involvement of duodenum and the initial part of jejunum; (2) detect the intestinal mesentery, the root of intestinal mesentery from upper left to lower right; (3) detect the surface of small intestine from the top-down.

#### Step 4: stomach and adjacent structures, omental bursa

Keep the supine position, (1) flop down the great omentum to subcolonic region to detect the serosa of anterior gastric wall and the greater curvature, evaluate the involved area; (2) lift the posterior gastric to assess the motility of tumor and posterior gastric wall, confirm the involvement of adjacent structures simultaneously. (3) Lift the left lobe of liver to detect the lesser curvature and the lesser omentum; (4) evaluate the distance between tumorous superior border and cardia; then, ascertain the involvement of pyloric ring and duodenal bulb; (5) detect the recessus of hepatorenalis; (6) if the tumor located in posterior gastric wall, open the left gastro-colic ligament about 3 cm and detect the omental bursa to evaluate whether there was tumor deposit in this region. (7) If there were suspicious tumor deposit, protractor biopsy by laparoscopic scissors and hemostasis was performed. (8) Suck and collect the peritoneal lavage in hepatorenal recess and splenic recess. (9) Finish the course of LE and suture peritoneal muscular sheath layers of porthole below umbilicus.

### Statistical processing and video clips

The categorical variables were shown as number and percentage, and continuous variables were described as median or mean number. The above evaluation was performed by Excel 2016 (Microsoft Office) and the SPSS version 22.0 (SPSS, Inc., Chicago, IL). The video clips were performed by Adobe Premiere Pro CS6.

## Results

### Patients

**Fig. 1:**
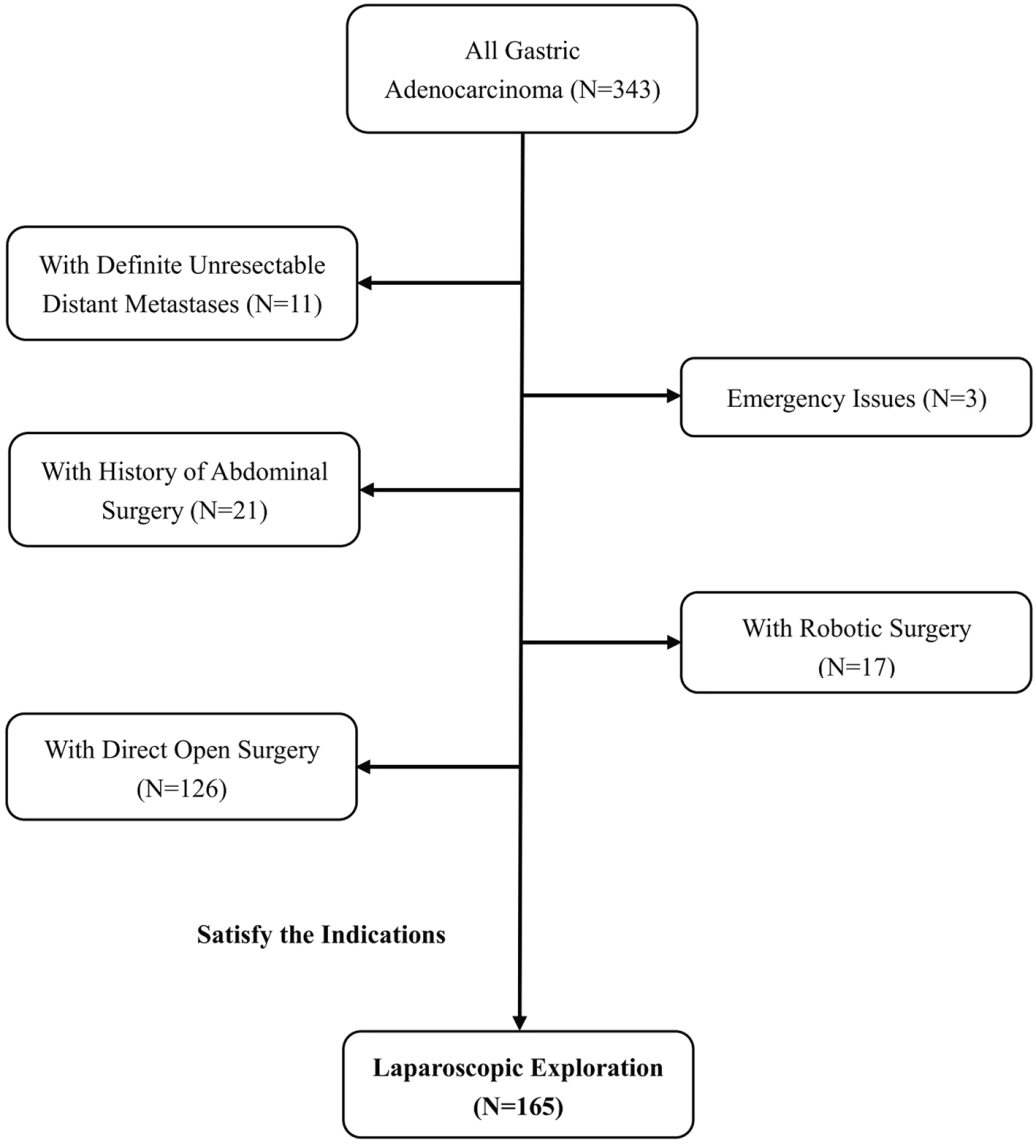
Flowchart of this study

We applied “Four-Step Procedure” LE to gastric adenocarcinoma since July 2016. From July 2016 to December 2017, 343 patients with gastric adenocarcinoma were admitted to a professional gastric cancer group in Gastrointestinal Surgery Department of West China Hospital. Among these 343 patients, there were 11 (3.2%) patients that without any surgical intervention since definite unresectable distant metastases on imaging examination. There were 150 (43.7%) patients only underwent open surgery since 21 patients with history of abdominal surgery, 3 patients with emergency issue and 126 patients acceped direct open surgery. There were 17 (5.0%) patients underwent robotic surgery. The flow diagram of evaluation is shown in Fig. 1.

### Clinicopathological characteristics

The enrolled patients had an average age of 59.1 years including 114 males and 51 females. The preoperative imaging staging is cT1 (20), cT2 (18), cT3 (37), cT4a (79), cT4b (11), cN0 (75), cN+ (90). There were seven (4.2%) patients with high suspicion of PD on the preoperative image. By the preoperative work-up, 39 (23.6%) patients were found with mild ascites. The clinicopathological features of cases in our study are indicated in Table 1.

**Table 1:** Demographics of patients who underwent surgical exploration in our study

Clinicopathological features	LE (*N* = 165) *N* (%)	Without LE (*N* = 167) *N* (%)
Age	59.1 ± 10.0	63.1 ± 11.6
Gender		
Male	114 (69.1%)	113 (67.7%)
Female	51 (30.9%)	54 (32.3%)
Tumor size	6.7 ± 3.3	4.9 ± 2.9
Macroscopic types		
0	20 (12.1%)	36 (21.6%)
1	0 (0%)	5 (3.0%)
2	75 (45.5%)	64 (38.3%)
3	63 (38.2%)	58 (34.7%)
4	7 (4.3%)	4 (2.4%)
cT^a^		
T1–2	38 (23.0%)	45 (26.9%)
T3	37 (22.4%)	46 (27.5%)
T4a	79 (47.9%)	60 (35.9%)
T4b	11 (6.7%)	16 (9.6%)
cN^a^		
N0	75 (45.5%)	79 (47.3%)
N+	90 (54.5%)	88 (52.7%)
Ascites^a^		
Ascites (−)	126 (76.4%)	142 (85.0%)
Ascites (+)	39 (23.6%)	25 (15.0%)
sP^b^		
P0	146 (88.5%)	161 (96.4%)
P1a	6 (3.6%)	3 (1.8%)
P1b	11 (6.7%)	1 (0.6%)
P1c	2 (1.2%)	2 (1.2%)
Cytological examination		
CY0	148 (89.7%)	156 (93.4%)
CY1	17 (10.3%)	11 (6.6%)
cH^a^		
H0	164 (99.4%)	165 (98.8%)
H1	1 (0.6%)	2 (1.2%)

^a^The c stage and ascites were indicated by preoperative CT scan

^b^The sP status was evaluated by surgical exploration

### The surgical parameters of “Four-Step Procedures” LE

The 165 cases were performed with LE all according to “Four-Step Procedures”; among them, 66 (40.0%) patients merely underwent LE without any further surgical intervention. A median duration of merely LE was 50.0 min that included time awaiting outcomes of frozen section diagnosis, and a median bleeding amount was 5.0 ml. For merely LE, most patients were discharged with no complications, only two (3.0%) patients occurred complications: one patient experienced intraoperative complication (diaphragmatic injury) and one experienced postoperative complication (pulmonary infection) that were both received medical treatment (Table 2).

**Table 2:** Surgical parameters of “Four-Step” LE

*N* = 66	*N* (%)
Duration (min)	50.0 (20–204)
Blood loss (ml)	5.0 (2–30)
Postoperative hospital stay (days)	6.0 (3–12)
Mortality	0 (0.0%)
Perioperative complications	2 (3.0%)
Pulmonary infection	1 (1.5%)
Diaphragmatic injury	1 (1.5%)

### The outcomes of “Four-Step Procedures” LE for P1 and/or Cy1

**Fig. 2:**
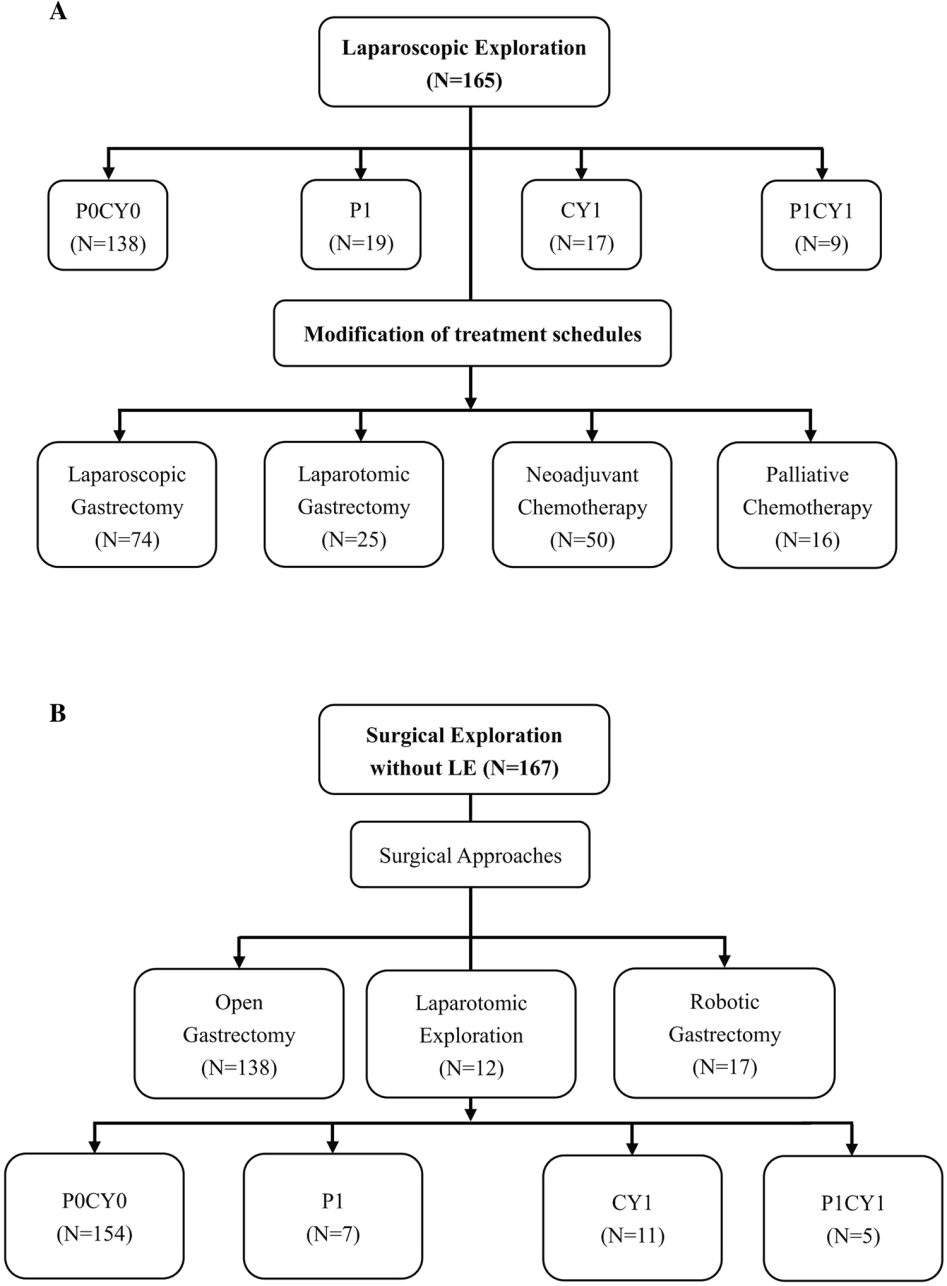
Results of patients underwent surgical exploration. **A** The results of LE. **B** The results of cases without LE

All of 165 patients were performed with LE according to “Four-Step Procedure”. After LE, total of 27 (16.4%) patients were found with P1 and/or Cy1 in our study: there were 19 (11.5%) patients proved with P1 (P1a:6, P1b:11, P1c:2), 17 (10.3%) patients confirmed with Cy1 and 9 (5.5%) patients with P1Cy1 (Fig. 2A). After stratified analysis, there was 0 (0.0%) patient with P1 and/or Cy1 in stages cT1–cT2, 1 (2.7%) patient with P1 and 1 (2.7%) patient with Cy1 in stage cT3, 15 (19.0%) patients with P1 and 14 (17.7%) patients with Cy1 in stage cT4a, 3 (27.3%) patients with P1 and 2 (18.2%) patients with Cy1 in stage cT4b. There were three (4.0%) patients with P1 and two (2.7%) with Cy1 in stage cN0, 16 (17.8%) patients with P1 and 15 (16.7%) patients with Cy1 in stage cN+. For the 39 patients with mild ascites on preoperative image: 10 (25.6%) patients with P1 and 9 (23.1%) patients with Cy1 (Table 3). There were 4 (57.1%) patients demonstrated with P1 in 7 patients with high suspicion of PD in our study.

**Table 3:** Stratified analysis outcomes by cT stage, cN stage after LE

cT stage	P1 *N* = 19	Cy1 *N* = 17	P1Cy1 *N* = 9
cT1 (*n* = 20)	0 (0.0%)	0 (0.0%)	0 (0.0%)
cT2 (*n* = 18)	0 (0.0%)	0 (0.0%)	0 (0.0%)
cT3 (*n* = 37)	1 (2.7%)	1 (2.7%)	0 (0.0%)
cT4 (*n* = 90)	18 (20.0%)	16 (17.8%)	9 (10.0%)
cT4a (*n* = 79)	15 (19.0%)	14 (17.7%)	8 (10.1%)
cT4b (*n* = 11)	3 (27.3%)	2 (18.2%)	1 (9.1%)
cN0 (*n* = 75)	3 (4.0%)	2 (2.7%)	2 (2.7%)
cN+ (*n* = 90)	16 (17.8%)	15 (16.7%)	7 (7.8%)
Mild ascites (*n* = 39)	10 (25.6%)	9 (23.1%)	5 (12.8%)

### The treatment disposition after “Four-Step Procedures” LE

Total of 165 patients were performed with “Four-Step procedure”, all the patients had been reconsidered consequently therapeutic regimens in terms of the outcome of LE: 50 (30.3%) patients received neoadjuvant chemotherapy (NACT) with 3–4 cycle Xelox regimen and 16 (9.7%) patients received palliative chemotherapy without any further surgical intervention, 74 (44.8%) patients underwent laparoscopic assistant gastrectomy and 25 (15.2%) patients transfer to open gastrectomy sequentially. Total of 66 (40.0%) patients changed their therapeutic schedule when compared with traditional treatment model (Fig. 2A). Among 27 patients with P1 and/or Cy1 after LE, 16 patients accepted palliative chemotherapy and 11 patients accepted NACT. Total of 167 patients without LE in our study, the results of these patients are indicated in Fig. 2B.

## Discussion

As we all know, the treatment model for gastric cancer had changed tremendously during the past decades which was illustrated by many previous researches [26, 27]. The multimodal treatment underlying surgical resection had become an inevitable tendency for the treatment of gastric cancer. The treatment regimens for gastric cancer also became more and more diversified such as surgical resection, NACT and biological immunotherapy [28–30]. When confronted with these options, an accurately preoperative staging for gastric cancer was eagerly demanded. LE for gastric cancer was primarily indicated for cases with gastric cancer that may have high risk of PD. The preoperative work-up like CT and GUS has some limitations on the identification for PD and cytology [31, 32]. LE was a minimally invasive and effective technique to acquire an accurate clinical stage for gastric cancer, especially for nubilous distant metastasis on imaging examinations and avoiding an unnecessary laparotomy in patients with gastric cancer [8–10]. In a word, LE might play an important role in the confirmation of PD and Cy stage among gastric cancer patients. For this reason, LE attracted more and more attention of surgeons and had become a focal topic.

However, the quality of LE would impact the accuracy for detecting PD. A steadfast manipulative rule is very critical, when considered to the concretely manipulative processes, different reports might have different explorative sequence and requirement [20, 24]. In our institution, we summarized the “Four-Step Procedure” for the execution of LE based on our retrospectively experience analysis.

Our “Four-Step Procedure” LE was obedient to the principle of hierarchy, comprehensive, logicality and “no-touch”. Our LE programs include entire detection of visceral surfaces, peritoneum, omentum, mesentery, parietal, lacuna and tumor location, depth, size and adjacent structures. The first step was performed under one Trocar with no alteration of position according to “O” model for anterior abdominal parietal and “S” model for visceral surfaces. All the locations were observed with perspective vision and close shot, respectively, this design and sequence effectively avoid omitting any scene. This step must pay attention to unnecessary side injury of small intestine, colon or livers. The second step was operated under three trocars with the position alteration; the main aims were to detect pelvic floor and collect peritoneal lavage for examination. In this process, the injury of upper rectum and bladder should be vigilant. The third step was performed after recovery of position, and the main purpose was to confirm the circumstance of mesentery and small intestine. This step should advert omental and mesenteric blood vessel. The last step should comply with “no-tough” criterion to detect primary lesion and the involved extent. We must protect the left liver, duodenum and spleen from damaging. For the suspicious tumor deposit, the locations, numbers, texture must be record and the frozen biopsy should be performed immediately. The peritoneal lavage should be reserved rationally and examined by the pathologist as soon as possible in case of cytolysis.

The operation procedure of LE was relative simple and convenient, and the operation time was short. The rate of morbidity among LE alone was also very low (0.6–5.1%) and with short in-hospital days [24, 31]. In our study, the postoperative complication was just 3.0% that was consistent with previous reports. Postoperative in-hospital day was 5 days including the first cycle chemotherapy. “Four-Step” LE might not increase morbidity and in-hospital days with a fast recovery after surgery. Above all, LE was very safe and feasible in clinical management, especially for gastric cancer before resection.

The main purpose of LE in gastric cancer was to confirm the PD to avoid pointless laparotomy. Our “Four-Step” procedure detected all potential locations of tumor deposits to avert overlook suspicious scene. Combined with the magnified view advantage of laparoscopy, many puny tumor deposits could be detected by LE. The previous reports indicated that false negative rate of LE ranged from 10.6 to 17.5% [24, 32]. The former study had indicated that most negligible locations were the mesentery of the small bowel, the transverse colon and inside of the bursa during LE [24]. Our “Four-Step Procedure” of LE routinely detected all above locations and intensively evaluated the posterior region of stomach, the omental bursa was also routinely evaluated for tumor located in posterior gastric wall; these steps had no doubt that improve the positive rate of PD of LE and reduced the false negative rate. Many previous reports had also illuminated that LE could detect PD in about 13–22.6% of patients with advanced gastric cancer especially for T4 stage [33–35]. In our study, LE could discover PD with rate of 20.0% in cT4 stage. This proportion was relatively a little higher than some previous reports. These outcomes manifested that “Four-Step” LE might be more thorough and elaborative. Since there was no consolidated inclusion criterion for LE in gastric cancer, different institutions employed diverse criterions and manipulative procedures for LE. On the other hand, different researches might have different staging distributions result in different sample size. These reasons might result in different positive rate of PD. Our study defined patients with cT3/4 irrespective the lymph nodes metastases as the indication for LE, a proportion of patients with cT1/2 that plan to be proceeded with laparoscopic gastrectomy also conducted LE before resection. This indication might slightly extensive and cause a relative lower specificity and accuracy. From our study, the positive rate of PD in cT4 stage was significantly higher than cT1–cT3 (27.3% vs. 1.3%); therefore, we deemed that LE should focus on cT4 stage by preoperative work-up. With respect to the indication for LE, macroscopic types, the status of lymph nodes and the density of omentum on contrast-enhanced CT should also be taken into consideration comprehensively.

In recent years, the role of NACT on advanced gastric cancer was demonstrated by many researches. The NCCN guideline also recommend that NACT should be a preferred selection for patients with T2 or more stage. A relevant portion of patients with resectable advanced gastric cancer might benefit from NACT [27, 36]. When concern to these patients, LE not only retained the opportunity for the second surgical resection after NACT, but also not increased the difficulty of next resection. In term of integrated outcomes of LE and preoperative work-up, the optimal clinical decision could be made. In our study, there was 66 (40.0%) patients modified their therapeutic schedule after LE. These patients were delivered to receive NACT or translational therapy to decrease the tumor stage and hope to acquire more curative resection. By the means of NACT and/or translational therapy, a fairly proportion of these patients might obtain a better prognosis. Above all, the significance of LE should be popularized to more gastric cancer patients to make LE more widespread.

Seldom limitations were consisted in our study; firstly, this was a retrospectively with limited number of total even. Secondly, the indications of LE were varied from different institutions; in our study, the indication may be a little extensive more factors needed to be in consideration. Thirdly, the choice of treatment regimen may be influenced by socioeconomic status of respective family, part of patients might hesitate when confronted with so many options. The “LE–NACT–Surgical resection” was relatively costly and postpone the surgery, and this may make patients feel troublesome and worried about the tumor progress. For these reasons, the new concept was not accepted by partial patients even after sufficient explanation by us and lead to partial patients who satisfy the indications of LE were not enrolled in this cohort.

## Conclusions

In conclusion, West China Hospital “Four-Step Procedure” of LE in our institution was safe and feasible for detecting unconspicuous metastasis in patients with gastric cancer. LE should be recommended in gastric cancer patients with cT3–4 stage for preoperative evaluation. LE might be also an effective and comprehensive technique to detect PD for gastric cancer in order to acquire an accurate preoperative stage and an optimal treatment regimen.

## Electronic supplementary material

Below is the link to the electronic supplementary material.

Supplementary Video 1 (MOV 164767 KB)
